# Non-atherosclerotic vasculopathies in stroke: a pathobiology-based framework

**DOI:** 10.3389/fneur.2026.1908716

**Published:** 2026-10-26

**Authors:** Archita Makharia, Divyani Garg, Achal Kumar Srivastava, Ayush Agarwal

**Affiliations:** Department of Neurology, All India Institute of Medical Sciences, New Delhi, India

**Keywords:** PCNSV, RCVS, stroke, vasculopathy, vessel wall MRI, young stroke

## Abstract

**Background:**

Non-atherosclerotic vasculopathies (NAV) are important causes of stroke, particularly in younger adults, but remain difficult to classify due to overlapping mechanisms and imaging features.

**Objectives:**

This study aimed to propose a three-compartment framework that organises NAV according to the dominant biological process underlying the observed vascular phenotype: structural arterial abnormalities, functional dysregulation of vascular tone or autoregulation, and immune- or infection-mediated vascular injury.

**Methods:**

We conducted a clinically oriented narrative review of PubMed and Embase, focusing on publications from 2015 onwards and prioritising guidelines, systematic reviews, major observational studies, and randomised trials where available. For this review, NAV were defined as selected cerebrovascular disorders in which a non-atherosclerotic abnormality of arterial structure, vascular tone or autoregulation, or immune- or infection-mediated vascular injury is central to the neurological phenotype.

**Results:**

NAV can be conceptualised into structural, functional, and inflammatory compartments. Structural disorders involve arterial wall pathologies, functional disorders reflect dysregulation of vascular tone, and inflammatory disorders involve immune-mediated vascular injury. Imaging, particularly vessel wall magnetic resonance imaging, provides supportive differentiation, although interpretation requires clinical correlation. The framework is intended to complement, rather than replace, aetiological and imaging-based classifications by linking vascular phenotypes to mechanism-directed diagnostic testing and initial management.

**Conclusion:**

A dominant-mechanism framework may help clinicians structure the evaluation of NAV and align diagnostic work-up and early management with the most likely underlying biology.

## Introduction

Atherosclerosis remains a leading cause of stroke worldwide; however, in young adults (defined as age <45 to <50 years in different studies), NAV represent an important and distinct cause of stroke (1–3). Cervical artery dissection is the most common, accounting for approximately 20–25% of cases (2). Similar stroke mechanisms, including artery-to-artery embolism, may occur in both atherosclerotic and NAV. However, NAV tend to occur in younger patients, are associated with fewer traditional vascular risk factors, and demonstrate lower markers of systemic atherosclerosis such as carotid intima-media thickness and homocysteine levels (4). These disorders remain challenging to recognise because their clinical presentations and radiological features often overlap, creating substantial diagnostic uncertainty (5). Although these disorders are particularly important in young adults and in cryptogenic stroke, the scope of NAV is not restricted by age. Some conditions, particularly cerebral amyloid angiopathy (CAA), predominantly affect older adults but remain relevant because the primary vascular pathology is non-atherosclerotic (6).

Despite the increasing recognition of NAV as important causes of stroke, their conceptual organisation remains fragmented. Existing classification systems categorise these disorders according to aetiology, affected vessel size, clinical presentation, or angiographic appearance (Table 1) (5, 7, 8). Although useful descriptively, these approaches have important limitations. First, the same disorder may fall into multiple categories depending on the framework used; for example, moyamoya disease may be viewed as a genetic arteriopathy, a large-vessel steno-occlusive disorder, or as an angiographic syndrome. Second, a recurring diagnostic challenge in NAV is their misclassification as atherosclerotic disease when interpretation relies primarily on luminal stenosis. Key imaging features that help distinguish these entities are summarised in Table 2. Third, phenotype-based classifications often provide limited guidance for management because disorders that look similar radiologically may require different evaluation and treatment strategies. Fourth, many NAV are dynamic processes, with temporal evolution of vascular abnormalities, such as resolution of vasoconstriction in RCVS or progression and healing of arterial dissection, which is not captured by static classification systems (8). For example, primary central nervous system vasculitis (PCNSV) and RCVS may both present with multifocal arterial narrowing despite markedly different underlying biology. Isolated middle cerebral artery disease may represent early or incomplete forms of moyamoya disease, highlighting the potential for misclassification as intracranial atherosclerosis in younger patients (4). As a result, clinicians are often left with parallel descriptive frameworks that categorise disease but do not adequately unify it.

**Table 1:** Common approaches to the classification of NAV.

Classification approach	Organising principle	Representative categories or examples	Strengths	Limitations
Aetiology-based	Presumed or established cause of vascular injury	Genetic or inherited; inflammatory or autoimmune; infectious; traumatic; connective-tissue related; haematological; treatment-related; idiopathic	Clinically intuitive; directs cause-specific investigations and treatment when the aetiology is established	The cause may initially be unknown; several mechanisms may coexist; broad categories can obscure the vascular phenotype
Vessel calibre and distribution	Predominant size and anatomical distribution of affected arteries	Large-, medium-, or small-vessel involvement; intracranial, extracranial, or multilevel disease	Helps localise disease and select appropriate imaging and tissue-based investigations	Many disorders affect multiple vessel sizes or territories; calibre and distribution do not establish the underlying mechanism
Clinical phenotype	Presenting syndrome, clinical tempo, and associated manifestations	Young stroke; recurrent thunderclap headache; recurrent transient ischaemic attacks; haemorrhagic stroke; headache with focal deficits; progressive or relapsing deficits	Supports rapid bedside pattern recognition and prioritisation of urgent differential diagnoses	Clinical syndromes are overlapping and non-specific; the same disorder may present with different phenotypes
Luminal or angiographic pattern	Morphology and temporal evolution of the arterial lumen	Focal or multifocal stenosis; segmental narrowing and dilatation; tapered occlusion; pseudoaneurysm; progressive steno-occlusion; collateral networks; moyamoya pattern	Widely available and useful for defining vascular distribution, severity, progression, and reversibility	Similar luminal appearances occur across mechanistically distinct disorders; angiographic patterns alone are rarely diagnostic
Vessel wall pathology or imaging phenotype	Predominant mural abnormality demonstrated histologically or suggested by vessel wall imaging	Intramural haematoma; intimal or medial abnormality; amyloid deposition; mural thickening or remodelling; concentric, eccentric, or absent enhancement	Provides information beyond luminal morphology and may narrow the differential diagnosis when integrated with clinical and laboratory findings	Imaging findings are affected by technique, disease stage, and treatment; enhancement and thickening are not mechanism-specific and histological confirmation is uncommon
Proposed dominant-biological-process framework	Biological process considered to predominate in the observed cerebrovascular phenotype	Structural abnormality of arterial architecture, integrity, or remodelling; functional dysregulation of vascular tone, endothelial function, or autoregulation; immune- or infection-mediated vascular injury; mixed or uncertain process	Links overlapping vascular phenotypes to discriminating investigations and provisional mechanism-directed management; accommodates overlap and evolution over time	A conceptual clinical aid rather than a validated diagnostic classification; compartment assignment may remain uncertain or change with disease stage and emerging evidence

**Table 2:** NAV that mimic atherosclerosis on imaging: key differentiating features and caveats.

Condition	Atherosclerosis-like imaging pattern	Imaging clues favouring NAV	Key caveat
Arterial dissection	Focal stenosis, tapered narrowing, or occlusion	Intramural hematoma on fat-suppressed T1; eccentric wall abnormality; pseudoaneurysm; increased external diameter	Classic luminal signs such as intimal flap or double lumen may be absent, especially intracranially
Moyamoya disease (especially early adult disease)	Isolated intracranial stenosis, particularly MCA or distal ICA disease	Reduced outer vessel diameter (constrictive remodelling); bilateral progression; collateral formation on follow-up	Early adult disease may lack classic distal ICA involvement or obvious moyamoya collaterals
FMD	Focal stenosis that may resemble plaque-related narrowing	Mid-to-distal vessel involvement; multifocal “string-of-beads” appearance; relative sparing of typical proximal atherosclerotic sites	Focal subtype may lack the classic beaded appearance and be under-recognised
Carotid web	Mild focal carotid bulb abnormality interpreted as plaque	Shelf-like projection from the posterior carotid bulb wall, best seen on sagittal or oblique CTA reconstructions	May be missed on axial views alone or misread as small non-stenotic plaque
RCVS	Multifocal intracranial narrowing resembling diffuse atherosclerotic stenosis	Reversibility on serial imaging; minimal or absent vessel wall enhancement on VWI	Early angiography may be normal; repeat imaging may be needed
Inflammatory vasculopathy/PCNSV	Multifocal or diffuse intracranial stenosis	Concentric vessel wall thickening and enhancement; associated leptomeningeal or parenchymal enhancement	Concentric enhancement is supportive, not specific; overlap may occur with other vasculopathies
Radiation-induced vasculopathy	Progressive stenosis or occlusion resembling accelerated atherosclerosis	Distribution corresponding to prior radiation field; delayed progressive changes; moyamoya-like collateralisation in some cases	Often recognised only when prior treatment history is actively sought

**NAV, Non-atherosclerotic vasculopathy; MCA, Middle cerebral artery; ICA, Internal carotid artery; CTA, Computed tomography angiography; RCVS, Reversible cerebral vasoconstriction syndrome; VWI, Vessel wall imaging; PCNSV, Primary central nervous system vasculitis.

## Methods

This narrative review was informed by searches of PubMed/MEDLINE and Embase from 1 January 2015 to 15 March 2026. Contemporary literature published from 2015 onwards was prioritised to reflect current diagnostic and therapeutic practice; however, this was not applied as a strict date restriction. Earlier publications were retained when they represented landmark clinical trials, foundational descriptions of disease mechanisms or phenotypes, established diagnostic criteria, or evidence not superseded by more recent studies. Search concepts included combinations of terms relating to “non-atherosclerotic vasculopathy,” “cerebral arteriopathy,” “arterial dissection,” “fibromuscular dysplasia,” “carotid web,” “moyamoya,” “reversible cerebral vasoconstriction syndrome,” “posterior reversible encephalopathy syndrome,” “central nervous system vasculitis,” “infection-associated arteriopathy,” “cerebral amyloid angiopathy,” “monogenic cerebral small-vessel disease,” “metabolic vasculopathy,” and relevant imaging, diagnostic, and treatment terms. Reference lists of pertinent reviews, guidelines, consensus statements, and included articles were also examined to identify additional studies. Three authors (AA, AM, and DG) collaboratively reviewed the retrieved literature and selected publications for inclusion on the basis of their relevance to pathobiology, imaging differentiation, diagnostic evaluation, and treatment. Differences regarding the relevance or inclusion of individual publications were resolved through discussion and consensus among these authors.

Because this was a narrative review intended to develop a clinically applicable conceptual framework rather than a formal systematic review, studies were selected on the basis of relevance to pathobiology, imaging differentiation, diagnostic work-up, and treatment implications. No formal evidence-grading framework was applied since this was a narrative review. The evidence supporting diagnostic and therapeutic statements was characterised according to its underlying study design, including randomised trials, systematic reviews and meta-analyses, observational cohorts, case series, translational studies, guidelines, and expert consensus. Where recommendations were derived from guidelines or consensus statements, this was stated explicitly. Given the rarity and heterogeneity of many NAVs, much of the available evidence was observational and was interpreted in light of the limitations of the underlying studies.

For this review, “non-atherosclerotic vasculopathy” was defined as a cerebrovascular disorder in which an abnormality of arterial structure or integrity, vascular tone or autoregulation, or immune- or infection-mediated vascular injury is central to the neurological phenotype. Disorders were selected when cerebrovascular involvement was an established manifestation and recognition of the underlying mechanism could influence diagnostic testing, surveillance, or treatment. Systemic, genetic, haematological, and metabolic diseases were included only when cerebrovascular involvement represented a recognised and clinically relevant component of the disease.

We excluded conventional atherosclerotic cerebrovascular disease; primary cardioembolic disorders; cerebral venous thrombosis and other primary venous diseases; isolated thrombophilia or hypercoagulable states without intrinsic cerebrovascular pathology; and common hypertensive, diabetic, or age-related cerebral small-vessel disease without a distinct arteriopathy. Intracranial aneurysms and vascular malformations were also outside the scope unless discussed as complications or differential diagnoses of an included vasculopathy. Systemic metabolic disorders are not included merely because they increase stroke risk. Fabry disease and MELAS are discussed because cerebrovascular dysfunction is a recognised component of their neurological phenotype and may involve non-atherosclerotic endothelial, smooth muscle, small-vessel, or autoregulatory mechanisms. The review was not intended to encompass every systemic disorder associated with stroke or endothelial dysfunction; the included conditions were selected as clinically relevant and representative examples of the proposed mechanistic framework rather than as an exhaustive catalogue.

### Pathobiological framework overview

NAVs may be better understood conceptually according to the biological processes underlying their cerebrovascular manifestations. Emerging advances in high-resolution vessel wall imaging (VWI) have enabled direct visualisation of arterial wall pathology, providing insights beyond luminal appearance and supporting a transition toward mechanism-oriented classification (8). We therefore outline a three-compartment pathobiological framework in which NAVs are grouped according to the dominant biological process underlying the observed vascular phenotype: structural abnormalities of arterial architecture, integrity, or remodelling; functional dysregulation of vascular tone, endothelial function, or autoregulation; and immune- or infection-mediated vascular injury (Figure 1; Supplementary Table 1). Structural vasculopathies are dominated by fixed or progressive abnormalities of arterial architecture, integrity, extracellular matrix, or remodelling. Functional vasculopathies are dominated by potentially reversible dysregulation of vascular tone, endothelial function, or autoregulation without primary destructive vascular inflammation. Inflammatory vasculopathies are dominated by immune- or infection-mediated vascular injury involving the endothelium, vessel wall, or perivascular tissues.

**Figure 1:**
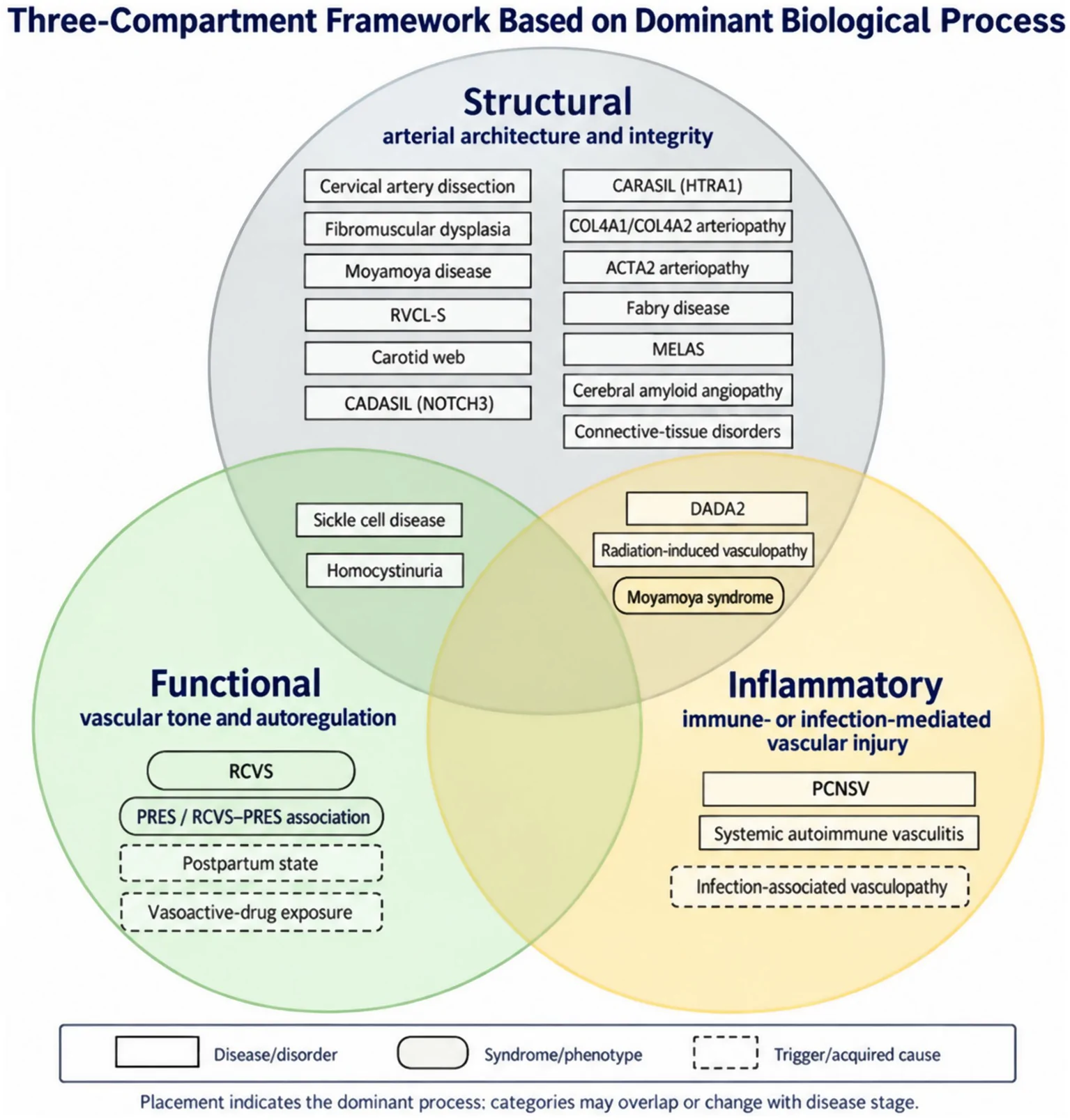
Three-compartment framework for NAV based on dominant biological process. Defined diseases, clinical or radiological syndromes, and acquired causes or triggers are distinguished using separate visual conventions. Placement indicates the biological process considered to predominate in the cerebrovascular phenotype rather than an exclusive or permanent classification. Some disorders may involve more than one compartment or shift between compartments according to disease stage and clinical context. RCVS and PRES are placed within the functional compartment because their association predominantly reflects dysregulation of vascular tone, endothelial function, and cerebral autoregulation; their coexistence does not, by itself, indicate primary vascular inflammation. RVCL-S, retinal vasculopathy with cerebral leukoencephalopathy and systemic manifestations; RCVS, reversible cerebral vasoconstriction syndrome; PRES, posterior reversible encephalopathy syndrome.

The framework is intended to complement established aetiological, vessel-size, syndromic, and imaging-based classifications. Its distinctive clinical question is not simply what the angiogram resembles, but which biological process is most likely to be driving the vascular phenotype and what should be tested or treated next. A predominantly structural hypothesis prioritises characterisation of mural architecture, remodelling, dissection, collateral formation, systemic vascular involvement, and inherited arteriopathy. A functional hypothesis prioritises trigger identification, serial vascular imaging, and demonstration of reversibility. An inflammatory hypothesis prioritises cerebrospinal fluid (CSF) examination, infectious and immune investigations, vessel wall imaging, and, in selected cases, biopsy.

The compartments are neither mutually exclusive nor fixed disease labels. Disorders with mixed pathophysiology are assigned according to the process predominantly responsible for the manifestation and clinical decision under consideration, and that assignment may change across disease stages. The framework should therefore be regarded as a clinical reasoning heuristic rather than a formally validated diagnostic taxonomy.

#### Structural vasculopathies: disorders of arterial architecture and integrity

Structural vasculopathies arise from intrinsic abnormalities of arterial wall architecture that compromise vascular integrity, resulting in stenosis, dissection, aneurysm formation, or altered remodelling, with subsequent ischaemic or haemorrhagic consequences (Supplementary Figure 1). Susceptibility genes such as *RNF213*, *NOTCH3*, *COL4A1*, *ACTA2*, *CTSA*, *KRIT1,* and *TREX1* have been implicated in arterial wall pathology (9). Histopathological insights reinforce this concept of vessel wall vulnerability: cervical artery dissection is characterised by intramural hematoma; fibromuscular dysplasia (FMD) is characterised by abnormal smooth muscle cell differentiation and medial fibroplasia, leading to disruption of elastic laminae, altered collagen deposition, and segmental arterial wall remodelling; and CADASIL is characterised by vascular smooth muscle degeneration with granular osmiophilic material deposition, leading to progressive remodelling and luminal narrowing (10–12).

Arterial dissection is an important structural cause of stroke in young adults. It may arise from an intimal disruption that permits luminal blood to enter the arterial wall or from primary intramural haemorrhage, potentially originating from the vasa vasorum, without an identifiable communication with the lumen. The resulting intramural hematoma may produce arterial stenosis or occlusion and promote thrombus formation and distal embolism; intracranial dissections may additionally present with subarachnoid or intraparenchymal haemorrhage (13). In many patients, cervical dissections follow trivial mechanical triggers such as minor neck rotation, coughing, or chiropractic manipulation, highlighting that underlying arterial wall fragility central to pathogenesis (12). It should be suspected in younger patients with sudden head or neck pain, connective tissue disease, minor trauma, or absence of conventional vascular risk factors, particularly when stenosis occurs at characteristic locations such as the intracranial vertebral artery, middle cerebral artery near the sphenoid ridge, or anterior cerebral artery near the falx (12). Reflecting this biological heterogeneity, structural vasculopathies may also be classified according to the predominant arterial wall component involved, as summarised in Table 3.

**Table 3:** Pathobiological subdivision of structural vasculopathies according to the dominant arterial wall component.

Dominant mural substrate	Representative disorders	Stroke mechanism
Intimal or medial smooth muscle architecture	Arterial dissection FMD Moyamoya disease *ACTA2*-related arteriopathy CADASIL	Artery-to-artery embolism Hemodynamic ischaemia Progressive stenosis Abnormal collateralisation
Extracellular matrix / connective tissue scaffold	Ehlers–Danlos syndrome type IV Marfan syndrome Pseudoxanthoma elasticum Arterial tortuosity syndrome (*SLC2A10*)	Dissection Aneurysm Rupture of fragile or abnormal arteries
Basement membrane	*COL4A1*/*COL4A2*-related disease	Small-vessel ischaemia Intracerebral haemorrhage Microbleeds
Degenerative mural protein deposition	CAA	Lobar intracerebral haemorrhage (micro- and macrohaemorrhages) Cortical superficial siderosis
Secondary acquired structural injury	Radiation-induced vasculopathy	Progressive stenosis Moyamoya-like arteriopathy Late vascular fragility

CAA-related inflammation and amyloid-β-related angiitis represent structural–inflammatory overlap syndromes.

FMD is associated with spontaneous cervical artery dissection, although its reported prevalence varies according to the diagnostic criteria used and the extent and modality of vascular screening (14). In the international STOP-CAD cohort of 3,714 patients with spontaneous cervical artery dissection, FMD was identified in 196 patients (5.3%). Compared with patients without FMD, those with FMD were less likely to have an isolated vertebral artery dissection (20.4% versus (vs.) 38.9%; adjusted OR 0.37, 95% CI 0.25–0.55). Multiple dissections were more frequent among patients with FMD in the unadjusted comparison (20.4% vs. 9.6%), although this association was attenuated after multivariable adjustment (adjusted OR 1.44, 95% CI 0.95–2.16). FMD was also associated with a higher 24-month risk of recurrent cervical artery dissection than was observed in patients without FMD (7.7% vs. 2.8%; adjusted HR 2.75, 95% CI 1.46–5.18), with 56.8% of recurrent events occurring within the first 3 months (14). In a separate multicentre cohort of patients with coexisting FMD and spontaneous cervical artery dissection, carotid involvement was more frequent than vertebral involvement (86% vs. 27%), and 33% of patients had multiple dissections (15). Conversely, among patients enrolled in the United States Registry for FMD, cervical artery dissection was a presenting manifestation in 12.1%, illustrating that the frequency of dissection among patients with established FMD addresses a different denominator from the prevalence of FMD among patients presenting with cervical artery dissection (16). Cervical artery dissection is a well-recognised cerebrovascular manifestation of FMD. In the US FMD Registry, patients also reported a history of transient ischaemic attack (13.4%), stroke (9.8%), amaurosis fugax (5.2%), intracranial aneurysm (approximately 7%), and subarachnoid haemorrhage (1.1%). These categories were not mutually exclusive, and the registry did not establish whether the ischaemic events occurred independently of dissection. Mechanistically, stroke and transient ischaemic attack in FMD are frequently related to cervical artery dissection, although artery-to-artery embolism or hypoperfusion associated with stenotic lesions may also contribute (10). Clinical manifestations are often non-specific, most commonly headache (≈60%, half migraine-like), along with pulsatile tinnitus (27.5%), neck pain, dizziness (20–26%), and isolated cervical bruit (22.2%), contributing to diagnostic delays that may extend 4–9 years (10).

Carotid webs are another under recognised vascular abnormality characterised by a thin, shelf-like intraluminal projection arising from the posterior wall of the carotid bulb. They are increasingly implicated as a cause of cryptogenic ischaemic stroke, particularly in young patients without traditional vascular risk factors, accounting for up to 13% of such cases in selected cohorts. The underlying mechanism relates to local flow disturbance and stasis along the web surface, promoting thrombus formation and subsequent artery-to-artery embolism (17).

Many structural vasculopathies are not confined to the cerebral circulation but represent manifestations of more systemic arterial disease. FMD may involve the renal and visceral arteries, while *COL4A1*-related disease may affect ocular and renal vasculature (10, 18). Similarly, *ACTA2*-related arteriopathy can produce thoracic aortic aneurysms (19). Recognition of this broader vascular involvement is clinically important, as it may prompt screening of extracranial arterial beds.

Cervical artery dissection may present as long-segment tapered stenosis, occlusion, pseudoaneurysm, or an eccentric narrowed lumen with increased external diameter, while VWI and fat-suppressed T1-weighted imaging can demonstrate a crescentic intramural hematoma. The characteristic “string-of-beads” appearance of FMD reflects alternating areas of stenosis and dilatation due to medial fibroplasia. According to the 2014 American Heart Association classification, FMD is angiographically categorised into multifocal and focal subtypes, although focal disease remains under recognised despite representing a distinct pathological entity (10). In contrast to atherosclerosis, which typically involves proximal arterial segments, FMD predominantly affects the mid-to-distal portions of the vessel (10). For carotid webs, CTA is the preferred initial imaging modality. Accurate detection of carotid webs requires careful evaluation on sagittal and oblique CTA reconstructions, as axial images alone may miss the characteristic shelf-like projection. They may often be missed or misinterpreted as atherosclerotic plaque, if careful evaluation of the posterior carotid wall is not performed. While MR angiography and ultrasound can aid in evaluation, their sensitivity is lower. Digital subtraction angiography may demonstrate a linear filling defect or shelf-like projection but is typically reserved for diagnostic uncertainty or pre-intervention planning (17).

Other structural vasculopathies have distinct imaging patterns. In moyamoya disease, progressive intracranial stenosis with the development of fragile collateral networks represents a characteristic imaging pattern, predisposing to both ischaemic and haemorrhagic complications, as these vessels are structurally fragile and prone to rupture, particularly in adult patients (20). In heritable connective tissue disorders such as Ehlers–Danlos syndrome, non-invasive vascular imaging is preferred, as invasive angiography carries increased risk due to arterial fragility (21). Increased arterial tortuosity, observed in connective tissue disorders, FMD, and monogenic arteriopathies, is considered a marker of arterial wall integrity (9, 10). However, the interpretation of VWI requires caution as technical and physiological factors (such as slow flow or vasa vasorum proliferation) may mimic pathological wall thickening or enhancement (8).

Although CAA differs from many other disorders discussed in this review because it predominantly affects older adults, it is included as a prototypical non-atherosclerotic small-arterial vasculopathy within the structural compartment because amyloid-β deposition within cortical and leptomeningeal vessel walls produces mural degeneration and vascular fragility. Blood-sensitive MRI sequences, particularly susceptibility-weighted imaging and T2*-weighted gradient-recalled echo imaging, may demonstrate the characteristic haemorrhagic markers of CAA, including strictly lobar cerebral microbleeds and cortical superficial siderosis (6). CAA-related inflammation (CAA-RI), characterised predominantly by perivascular inflammation, and amyloid-β-related angiitis (ABRA), characterised by transmural or granulomatous vasculitic inflammation, represent overlap between the structural and inflammatory compartments. These inflammatory forms commonly demonstrate asymmetric vasogenic oedema with leptomeningeal or parenchymal enhancement in addition to haemorrhagic markers of CAA. Their recognition is clinically important because they may resemble primary central nervous system vasculitis (PCNSV) but are potentially responsive to immunosuppressive treatment (22). This overlap further illustrates that the proposed compartments represent dominant mechanisms rather than mutually exclusive categories. Key imaging features that help distinguish structural from inflammatory vasculopathies are summarised in Table 4.

**Table 4:** Typical imaging tendencies and interpretive limitations in selected structural and inflammatory intracranial arteriopathies.

Imaging feature	Structural vasculopathies: typical tendency	Inflammatory vasculopathies: typical tendency	Diagnostic value, overlap, and modifiers
Dominant biological process	Abnormality of arterial architecture, integrity, extracellular matrix, or remodelling	Immune- or infection-mediated injury involving the endothelium, vessel wall, or perivascular tissues	Processes may coexist or evolve sequentially and are not anatomically discrete compartments
Luminal narrowing	May be focal, segmental, tapered, beaded, or progressively steno-occlusive	May be focal or multifocal and may involve vessels of different sizes and territories	Luminal morphology is not mechanism-specific. Dissection, FMD, RCVS, vasculitis, infection-associated arteriopathy, moyamoya angiopathy, and atherosclerosis may have overlapping appearances
Evolution on serial imaging	Often persistent or progressive; dissection-related abnormalities may heal or remodel	May improve, persist, progress, or recur according to disease activity and treatment	Reversibility favours a functional process such as RCVS but is not independently diagnostic. Findings must be interpreted with the clinical course and treatment received
Vessel wall enhancement	May be absent, focal, eccentric, or concentric depending on the disorder and disease stage	Smooth concentric enhancement is frequently described, although focal, eccentric, patchy, or absent enhancement may occur	Concentric enhancement supports but does not establish vasculitis. Similar enhancement may occur with atherosclerosis, dissection, moyamoya angiopathy, infection, radiation injury, recent infarction, or vascular intervention
Vessel wall thickening	May reflect intramural hematoma, fibrosis, matrix abnormality, or arterial remodelling and may be eccentric or concentric	May reflect inflammatory infiltration or oedema and may be concentric, eccentric, or irregular	Considerable overlap exists.
Arterial remodelling	Constrictive or expansive remodelling may occur; reduced outer arterial diameter may support moyamoya angiopathy, whereas mural expansion may accompany dissection	Acute inflammation may produce mural oedema; chronic inflammation may result in fibrosis, constrictive remodelling, or fixed stenosis	Remodelling may indicate the nature or chronicity of vascular injury but does not independently establish its aetiology
Collateral formation	May be prominent in chronic progressive steno-occlusive disorders, particularly moyamoya angiopathy	May also develop in chronic inflammatory or infection-associated steno-occlusive disease	Collateral formation primarily reflects the severity and duration of flow limitation rather than a uniquely structural mechanism
Associated imaging findings	Intramural hematoma, intimal flap, aneurysmal change, arterial tortuosity, reduced outer diameter, or characteristic collateral networks may provide diagnostic clues	Infarcts of different ages, haemorrhage, leptomeningeal enhancement, or other inflammatory abnormalities may coexist	Associated findings may refine the differential diagnosis but remain supportive and require correlation with clinical, CSF, laboratory, and angiographic findings
Response on follow-up imaging	Structural abnormalities may persist despite clinical stabilisation, although dissection-related findings may resolve or remodel	Enhancement or narrowing may diminish after treatment but may also persist despite clinical improvement	Change in enhancement may support altered inflammatory activity but is not a validated stand-alone marker of treatment response
Technical and temporal influences	Appearance may vary according to lesion chronicity, healing, and previous intervention	Enhancement may vary with inflammatory activity and immunosuppressive treatment	Findings are influenced by spatial resolution, field strength, sequence design, blood and CSF suppression, contrast timing, motion, partial-volume effects, disease stage, recent ischaemia, and previous treatment

**The features shown represent typical tendencies rather than diagnostic criteria. Considerable overlap occurs among structural, inflammatory, functional, infectious, and atherosclerotic arteriopathies. Vessel wall enhancement, including smooth concentric enhancement, is neither necessary nor sufficient for diagnosing vasculitis. Conversely, absent enhancement does not exclude inflammation, particularly in treated disease, small-vessel involvement, or vessels below the spatial resolution of VWI. Findings should be interpreted alongside the clinical tempo, CSF and laboratory results, luminal and parenchymal imaging, treatment history, and temporal evolution. CSF, Cerebrospinal fluid; FMD, Fibromuscular dysplasia; RCVS, Reversible cerebral vasoconstriction syndrome; VWI, Vessel wall imaging.

In the structural compartment, biomarker assessment is primarily directed toward identifying underlying abnormalities of the arterial wall, particularly involving genetic pathways and extracellular matrix integrity (Figure 2). Genetic testing currently has the greatest clinical utility, especially in suspected hereditary arteriopathies such as CADASIL, *COL4A1/2*-related disorders, and *ACTA2*-associated vasculopathy, where it can establish a definitive diagnosis and guide family screening (9). Beyond genetics, emerging biomarkers are increasingly focussed on pathways involved in arterial wall remodelling and structural integrity. Matrix metalloproteinases (MMPs), particularly MMP-2 and MMP-9, have been implicated in extracellular matrix degradation and vascular remodelling, contributing to arterial fragility and dissection (23). Transforming growth factor-β (TGF-β) signalling pathways, well described in connective tissue disorders such as Marfan syndrome, play a central role in extracellular matrix regulation and vascular smooth muscle function (24). Other candidate biomarkers include markers of endothelial dysfunction, inflammatory activation, and connective tissue turnover, although their clinical application remains investigational (25). These insights raise the possibility that future therapeutic strategies may target pathways involved in arterial wall remodelling and extracellular matrix integrity.

**Figure 2:**
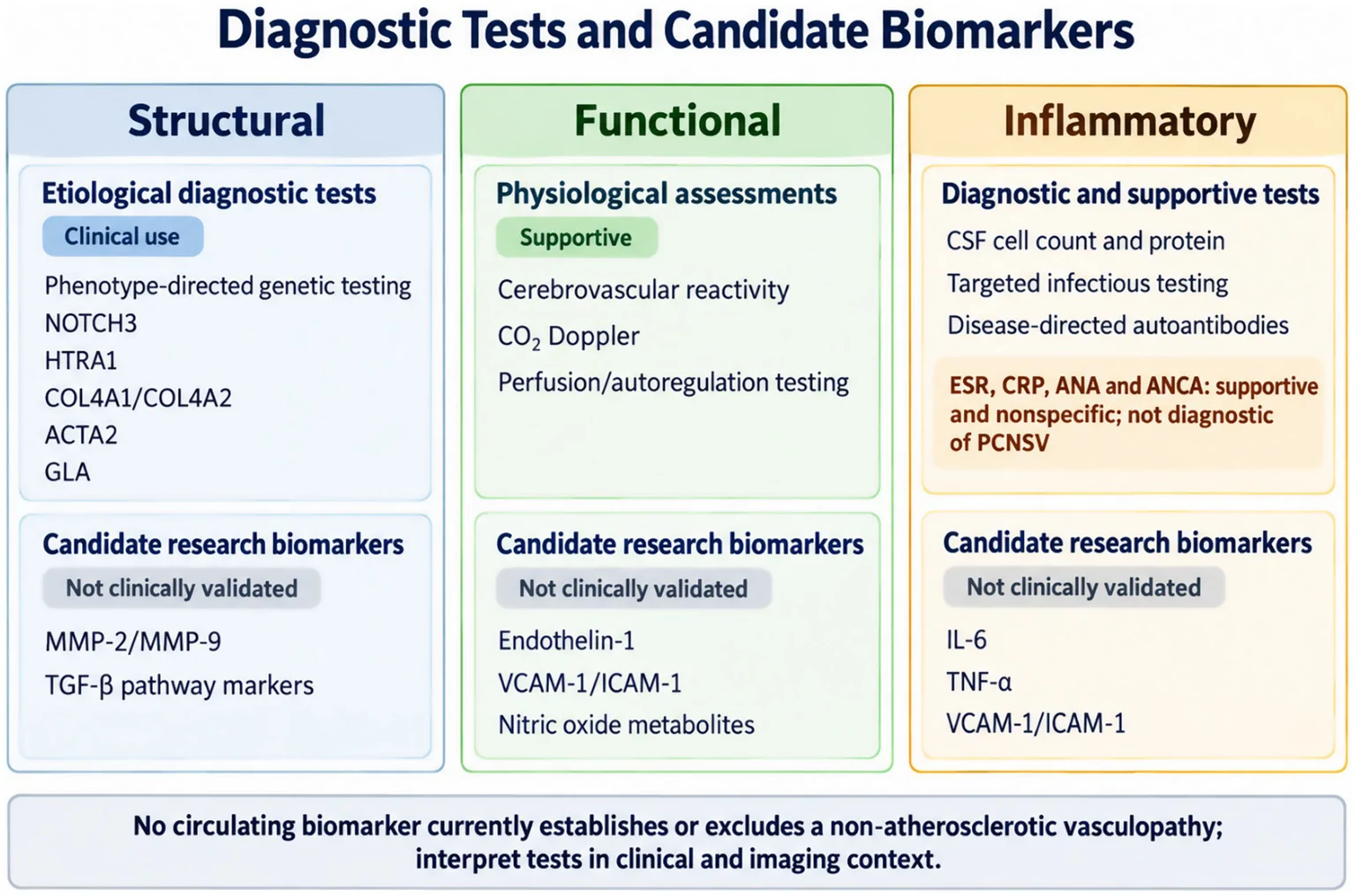
Diagnostic tests and candidate biomarkers. Tests are grouped as aetiological diagnostic tests, physiological assessments, non-specific supportive investigations, and candidate research biomarkers. Genetic testing may establish a specific inherited diagnosis when guided by the clinical phenotype. Cerebrovascular reactivity and CO_2_ Doppler are physiological assessments rather than biomarkers. ESR, CRP, ANA, and ANCA may support investigation for systemic inflammatory disease but are non-specific and do not establish PCNSV. The listed molecular markers remain investigational and are not clinically validated for diagnosing or excluding a non-atherosclerotic vasculopathy.

Therapeutic strategies in structural vasculopathies primarily target the hemodynamic and thromboembolic consequences of arterial wall disease rather than the underlying pathology. An individual-patient-data meta-analysis of randomised trials involving 444 patients with arterial dissection found no significant difference between anticoagulation and antiplatelet therapy for the 90-day composite outcome of ischaemic stroke, death, or major bleeding. Accordingly, the European Stroke Organisation guideline considers either strategy acceptable for cervical artery dissection (26–28). A systematic review and meta-analysis, based predominantly on non-randomised evidence, found no significant difference in ischaemic or haemorrhagic outcomes between vitamin K antagonists and direct oral anticoagulants (29). However, uncertainties persist regarding optimal treatment duration, management of persistent pseudoaneurysms, and the role of endovascular intervention. In symptomatic moyamoya disease, revascularisation is widely used on the basis of observational data and guideline recommendations, although questions remain regarding timing, patient selection, and the relative value of direct vs. indirect procedures (20). Patients with FMD may require counselling on physical activities, and expert consensus advises avoiding potential dissection triggers such as chiropractic cervical manipulation and high-acceleration rides, although these recommendations are based primarily on expert consensus and should be individualised according to vascular phenotype and clinical history (30). In patients with symptomatic carotid webs, antithrombotic therapy alone has been associated with a substantial risk of recurrent ischaemic stroke (31, 32). In one cohort, 17% experienced recurrent stroke within 2 years despite predominantly antiplatelet-based medical management (32). This risk may reflect persistent flow disturbance and thrombus formation within the web, with embolic potential rather than the degree of luminal stenosis determining clinical risk (17). A systematic review of 158 patients reported no recurrent strokes among the 72% who underwent carotid revascularisation, and a more recent meta-analysis found a substantially lower recurrence risk with carotid endarterectomy or stenting than with medical therapy alone (RR 0.11, 95% CI 0.06–0.28) (31, 33). Accordingly, carotid revascularisation may be considered when a carotid web is ipsilateral to an otherwise unexplained ischaemic event, following exclusion of competing stroke mechanisms and multidisciplinary assessment (17, 34). However, the available evidence is derived predominantly from observational studies, and randomised trials comparing medical therapy, carotid endarterectomy, and carotid artery stenting are lacking (17, 33). Inherited arteriopathies such as CADASIL and *COL4A1*-related disorders are largely managed with supportive care, vascular risk factor control, and prevention of secondary complications (9). Despite advances in understanding the molecular and structural basis of these disorders, disease-modifying therapies targeting the underlying arterial wall pathology are currently lacking.

Across structural vasculopathies, the unifying clue is that the vessel wall is intrinsically abnormal, so imaging interpretation should look beyond luminal stenosis to mural hematoma, remodelling pattern, external diameter, arterial tortuosity, collateral formation, and extracranial vascular involvement.

#### Functional vasculopathies: disorders of vascular tone and autoregulation

Functional vasculopathies are characterised by dynamic, often reversible changes in arterial calibre caused by dysregulation of vascular reactivity, sympathetic overactivity, endothelial dysfunction, and impaired cerebral autoregulation or neurovascular coupling (Supplementary Figure 2) (8, 35). Physiological studies, including transcranial Doppler and perfusion imaging, support impaired autoregulation, altered flow velocity responses, and abnormal cerebrovascular reactivity as key pathophysiological features (35, 36). Experimental evidence also suggests that vascular smooth muscle cells may undergo phenotypic switching under pathological conditions, altering responsiveness to vasoactive stimuli, although these mechanisms remain incompletely understood and are supported primarily by translational rather than clinical studies (37). Metabolic pathways involved in neurovascular coupling, including the lactate-GPR81 signalling axis, have also been proposed as modulators of cerebrovascular reactivity in selected metabolic or mitochondrial angiopathies (38).

RCVS, the prototypical functional vasculopathy, is characterised by recurrent thunderclap headaches over several days, often preceding neurological complications and offering a diagnostic window. Identifiable triggers are present in 50–60% of cases and include vasoactive drugs, the postpartum state, and illicit substances. In a contemporary systematic review of unselected RCVS cohorts, the pooled frequency of ischaemic stroke was 15.9% (95% CI 9.6–23.4%), while that of intracranial haemorrhage was 22.1% (95% CI 10.0–39.6%). However, frequencies varied widely across individual studies, reflecting substantial clinical and methodological heterogeneity (39). Haemorrhagic complications typically occur early, possibly related to sudden surges in blood pressure and failure of autoregulatory mechanisms, whereas ischaemic events are more commonly observed later, paralleling the temporal evolution of vasoconstriction (39, 40). Observational cohorts and systematic reviews suggest that clinical recovery is favourable in most patients, although severe vasoconstriction, early haemorrhage, and delayed diagnosis are associated with poorer outcomes; recurrence has been reported in approximately 5–10% of patients (35, 41).

Similar mechanisms are implicated in postpartum and drug-induced vasoconstriction, states of catecholamine excess and posterior reversible encephalopathy syndrome (PRES). RCVS and PRES may coexist and share abnormalities of cerebrovascular regulation, including endothelial dysfunction, impaired autoregulation, and altered vascular tone. This relationship is best understood as overlap between syndromes of cerebrovascular dysregulation rather than as evidence of primary vascular inflammation. RCVS therefore remains classified as a predominantly non-inflammatory functional vasculopathy unless an independent immune- or infection-mediated vascular process is demonstrated (42).

Imaging in functional vasculopathies typically shows multifocal arterial narrowing on angiography, reflecting heterogeneous dysregulation of vascular tone across arterial territories. A key feature is reversibility on follow-up imaging, making serial assessment more informative than a single static study. In RCVS, vasoconstriction may follow a centripetal pattern, beginning distally and progressing proximally, which may explain normal early imaging. Vasoconstriction usually peaks within 1–2 weeks and resolves over 1–3 months, making imaging timing critical. Observational imaging studies indicate that high-resolution VWI in RCVS generally demonstrates absent or minimal wall enhancement, although this finding is neither universal nor independently diagnostic and should be interpreted alongside the clinical presentation, CSF findings, and evolution on serial imaging. Prolonged or recurrent vasoconstriction may, however, lead to secondary vascular remodelling, highlighting overlap with structural mechanisms (5, 35, 41).

Distinguishing RCVS from inflammatory vasculopathies such as PCNSV is a key diagnostic challenge (Table 5). Although both may cause multifocal arterial narrowing, RCVS typically presents with recurrent thunderclap headaches and reversible vasoconstriction, whereas PCNSV more often causes subacute neurological deficits and progressive vascular abnormalities. Observational imaging studies suggest that prominent concentric vessel wall enhancement favours an inflammatory vasculopathy; however, this finding is supportive rather than definitive and may occur in other vascular disorders (43). No single imaging or laboratory feature is definitive in isolation; diagnosis rests on the combined pattern of onset, trigger context, CSF profile, vessel wall imaging, and reversibility on follow-up. The RCVS2 score has been proposed as a clinical tool to distinguish RCVS from PCNSV (44). This distinction is clinically critical, as misdiagnosis may lead to inappropriate immunosuppressive therapy.

**Table 5:** Key differences between RCVS and PCNSV.

Feature	RCVS	PCNSV	Important caveat
Clinical presentation and evolution	Acute onset with recurrent thunderclap headaches over days to weeks; focal deficits, seizures, or encephalopathy may occur	Usually subacute or progressive headache, encephalopathy, seizures, or multifocal deficits; acute presentations are possible	Clinical tempo is highly informative but not independently diagnostic
CSF	Usually normal or near-normal; minor abnormalities may occur	Frequently shows mild pleocytosis or elevated protein, although normal CSF is possible	Marked pleocytosis or low glucose should prompt evaluation for infection or another diagnosis
Parenchymal MRI	May be normal or show convexity subarachnoid haemorrhage, ischaemic infarction, intracerebral haemorrhage, cerebral oedema, or PRES	May show multifocal infarcts of different ages, haemorrhage, tumour-like lesions, or leptomeningeal enhancement; susceptibility weighted imaging may reveal multiple microhaemorrhages; nodular contrast enhancement on T1 + C sequences	Considerable overlap exists; no single parenchymal pattern is diagnostic
Luminal imaging and temporal evolution	Multifocal segmental narrowing and dilatation; abnormalities may be absent early and generally improve substantially or resolve within 12 weeks	May show focal or multifocal stenosis, occlusion, dilatation, or beading; abnormalities may persist, progress, fluctuate, or improve with treatment	Angiographic beading is non-specific. Reversibility supports RCVS, but persistent narrowing alone does not establish PCNSV
Vessel wall MRI	Often absent or mild enhancement, although concentric or eccentric enhancement may occur	Concentric thickening and enhancement may support inflammation, but enhancement may be variable or absent	Enhancement pattern alone cannot diagnose vasculitis or exclude RCVS; findings are influenced by technique, vessel size, disease stage, and treatment
Role of biopsy	No routine role	Brain or leptomeningeal biopsy may be considered when diagnostic uncertainty persists, particularly in suspected small-vessel PCNSV	Sensitivity is limited by segmental disease; targeted biopsy may also identify important mimics
Initial management	Remove potential triggers and provide supportive care and careful blood-pressure management. Calcium-channel blockers may reduce thunderclap-headache burden, but prevention of ischaemic or haemorrhagic complications is unproven; glucocorticoids should generally be avoided	Exclude infection and other mimics before initiating glucocorticoids, with additional immunosuppressive therapy according to disease severity and course	Treatment should not be selected from angiographic or vessel wall imaging appearance alone

The listed features represent typical tendencies rather than diagnostic criteria. RCVS and PCNSV may overlap clinically and radiologically. Diagnosis should integrate clinical tempo, exposures, CSF findings, parenchymal and vascular imaging, and serial evolution. CSF, cerebrospinal fluid; PCNSV, primary central nervous system vasculitis; PRES, posterior reversible encephalopathy syndrome; RCVS, reversible cerebral vasoconstriction syndrome.

No biomarker is currently established for routine diagnosis of functional vasculopathies, and proposed markers remain investigational (Figure 2) (25, 36).

Management of RCVS is based primarily on withdrawal of vasoactive or sympathomimetic triggers, symptomatic treatment, and careful management of blood pressure; however, randomised treatment trials are lacking (45). Nimodipine is frequently used empirically for thunderclap headache. In a prospective observational cohort of 82 patients in which all participants received nimodipine, 71 patients (86.6%) experienced remission of thunderclap headache after treatment initiation, and earlier administration was associated with a shorter headache course (46). However, the absence of an untreated comparator precludes a causal inference regarding efficacy, and there is no established evidence that nimodipine prevents ischaemic stroke, intracranial haemorrhage, or other neurological complications. Glucocorticoids should generally be avoided in patients with RCVS unless required for a separate established indication. In a retrospective cohort of 162 patients, glucocorticoid exposure was independently associated with clinical, radiological, and angiographic worsening and with poorer outcomes, although the observational design does not establish causality (47).

#### Inflammatory vasculopathies: immune- or infection-mediated vascular injury

Unlike functional vasculopathies, in which luminal narrowing primarily reflects dynamic dysregulation of vascular tone or autoregulation without primary destructive vascular inflammation, inflammatory vasculopathies are driven predominantly by immune- or infection-mediated vascular injury involving the endothelium, vessel wall, or perivascular tissues. Endothelial activation, leucocyte infiltration, cytokine-mediated injury, and secondary thrombosis may produce luminal narrowing, impaired perfusion, and, in severe cases, vessel wall necrosis. Haemorrhagic complications may occur when inflammation compromises arterial wall integrity (48, 49). The term “inflammatory” is therefore used as a concise category label for this dominant biological process and does not denote a separate anatomical compartment.

These disorders encompass a heterogeneous spectrum, including PCNSV, systemic autoimmune vasculitis with secondary cerebrovascular involvement, infection-associated arteritis, and post-infectious or para-infectious arteriopathy. (Table 6) Not all infection-associated or post-infectious cerebrovascular syndromes represent histopathological confirmed vasculitis. Depending on the pathogen and clinical context, vascular injury may result from direct infectious invasion of the arterial wall, immune-mediated arteritis, endothelial dysfunction, systemic inflammation, or activation of prothrombotic pathways (50).

**Table 6:** Inflammatory vasculopathies organised by dominant immune-mediated mechanism.

Category	Representative disorders
PCNSV	PCNSV
Systemic autoimmune vasculitis	SLE, ANCA vasculitis, PAN, Behçet, GCA, and Takayasu
Infectious vasculitis	VZV, tuberculosis, syphilis, and HIV
Post-infectious arteriopathy	Post-varicella focal cerebral arteriopathy (FCA)

**PCNSV, Primary central nervous system vasculitis; SLE, Systemic lupus erythematosus; ANCA, Antineutrophil cytoplasmic antibody; PAN, Polyarteritis nodosa; GCA, Giant cell arteritis; VZV, Varicella-zoster virus; HIV, Human immunodeficiency virus.

Clinically, inflammatory vasculopathies often present with subacute or fluctuating neurological deficits, cognitive impairment, or multifocal stroke, frequently accompanied by systemic features. In practice, a subacute course combined with constitutional or autoimmune features should raise suspicion for an inflammatory mechanism. Within this spectrum, PCNSV is itself heterogeneous: large- and medium-vessel disease more often causes angiographic abnormalities and stroke, whereas small-vessel disease may present with cognitive impairment, seizures, inflammatory CSF findings, and biopsy-positive but angiography-negative disease. Histopathology may show granulomatous or lymphocytic inflammation. PCNSV remains diagnostically challenging because it overlaps with mimics such as RCVS, intracranial atherosclerosis, infection-related vasculopathy, and systemic inflammatory disease with CNS involvement (51).

Imaging findings reflect vascular inflammation but are not disease-specific. Conventional angiographic techniques may demonstrate multifocal arterial narrowing across vascular territories, while high-resolution VWI reveals concentric narrowing of the vessel wall with enhancement. Spin-echo-based black-blood approaches with dedicated blood and CSF suppression improve wall conspicuity and are the current practical standard in many centres. Leptomeningeal or parenchymal enhancement accompanying vessel wall enhancement increases the probability of vasculitis (52). These findings are supported by observational imaging studies but are not independently diagnostic because similar enhancement patterns may occur in non-inflammatory arteriopathies. Serial imaging may further aid diagnosis by demonstrating evolution with disease activity or response to therapy (8, 48, 53). Overdiagnosis of vasculitis is a major practical hazard, particularly when angiographic narrowing or non-specific vessel wall enhancement is interpreted without adequate clinical and CSF correlation.

Laboratory and CSF abnormalities may support an inflammatory mechanism, but their diagnostic specificity is limited, and the available evidence derives mainly from observational cohorts and case series (Figure 2) (54). Despite advances in imaging and laboratory evaluation, definitive diagnosis remains challenging. Brain and leptomeningeal biopsy remains the diagnostic reference standard for PCNSV, although its sensitivity is limited by sampling error arising from the patchy distribution of vascular inflammation; this limitation is documented primarily in observational series (49, 55).

Management is directed toward suppression of vascular inflammation, treatment of underlying causes, and prevention of secondary ischaemic or thrombotic complications. Corticosteroids are commonly used as first-line therapy, often in combination with additional immunosuppressive agents such as cyclophosphamide or other steroid-sparing therapies; however, supporting evidence is derived largely from retrospective observational cohorts and expert consensus, as randomised controlled treatment trials are lacking (56). In infection-associated vasculopathies, targeted antimicrobial therapy is essential. Early recognition and treatment are associated with improved outcomes.

### Mixed-mechanism and border-zone Vasculopathies

The three compartments describe dominant biological processes rather than mutually exclusive disease entities. Overlap is therefore expected and does not imply that every disorder must be forced into a single permanent category. The relevant classification depends on which process is predominantly responsible for the vascular manifestation being evaluated and which diagnostic or therapeutic decision is being made. Compartmental predominance may also evolve over time, for example, an initial endothelial or inflammatory injury may subsequently produce fixed fibrosis, remodelling, and structural stenosis (Figure 1).

Sickle cell disease, DADA2, radiation vasculopathy, infection-associated arteriopathy, and moyamoya syndromes illustrate this context-dependent assignment. Sickle cell disease may combine haemolysis-associated endothelial dysfunction, thrombosis, inflammation, and progressive arterial remodelling (57, 58). DADA2 has inflammatory, endothelial, and thrombotic manifestations (59, 60). Radiation vasculopathy may evolve from endothelial injury and inflammation to fibrosis, accelerated atherosclerotic change, and fixed structural stenosis, so its dominant compartment may differ according to the interval from radiation exposure and the vascular lesion being evaluated (61). Infection-associated arteriopathies may reflect direct vascular invasion, immune-mediated injury, endotheliopathy, thrombosis, or combinations of these processes (50). Moyamoya represents a distinctive progressive steno-occlusive arteriopathy and collateral vascular phenotype. A similar angiographic pattern may occur as moyamoya syndrome in association with genetic, inflammatory, haematological, infectious, or radiation-related conditions (20, 62).

The frequent coexistence of RCVS and PRES supports mechanistic overlap within the broader domain of cerebrovascular dysregulation, particularly endothelial dysfunction, impaired autoregulation, and vascular-tone instability. These mechanisms do not themselves establish primary vascular inflammation. RCVS therefore remains classified predominantly as a functional, non-inflammatory vasculopathy, while PRES is regarded as a related disorder of endothelial function and autoregulatory failure rather than evidence of functional–inflammatory overlap.

Infection-associated cerebrovascular injury may involve heterogeneous and overlapping mechanisms, including direct vascular invasion, immune-mediated inflammation, endothelial dysfunction, thrombosis, cardio embolism, and conventional vascular risk factors. COVID-19-associated stroke illustrates this mechanistic heterogeneity but is not considered a discrete post-COVID vasculopathy in this framework (50, 63). These conditions should therefore be mapped according to the process driving the current presentation rather than assigned solely according to their disease name.

## Clinical application of the framework

The proposed classification aligns with the growing emphasis on mechanism-based stroke evaluation but provides a simplified and clinically applicable structure integrating imaging, clinical features, and pathobiology. It can be applied in a pragmatic, stepwise manner in clinical practice and guide targeted diagnostic evaluation and management. A practical algorithm for this approach is shown in Figure 3.

**Figure 3:**
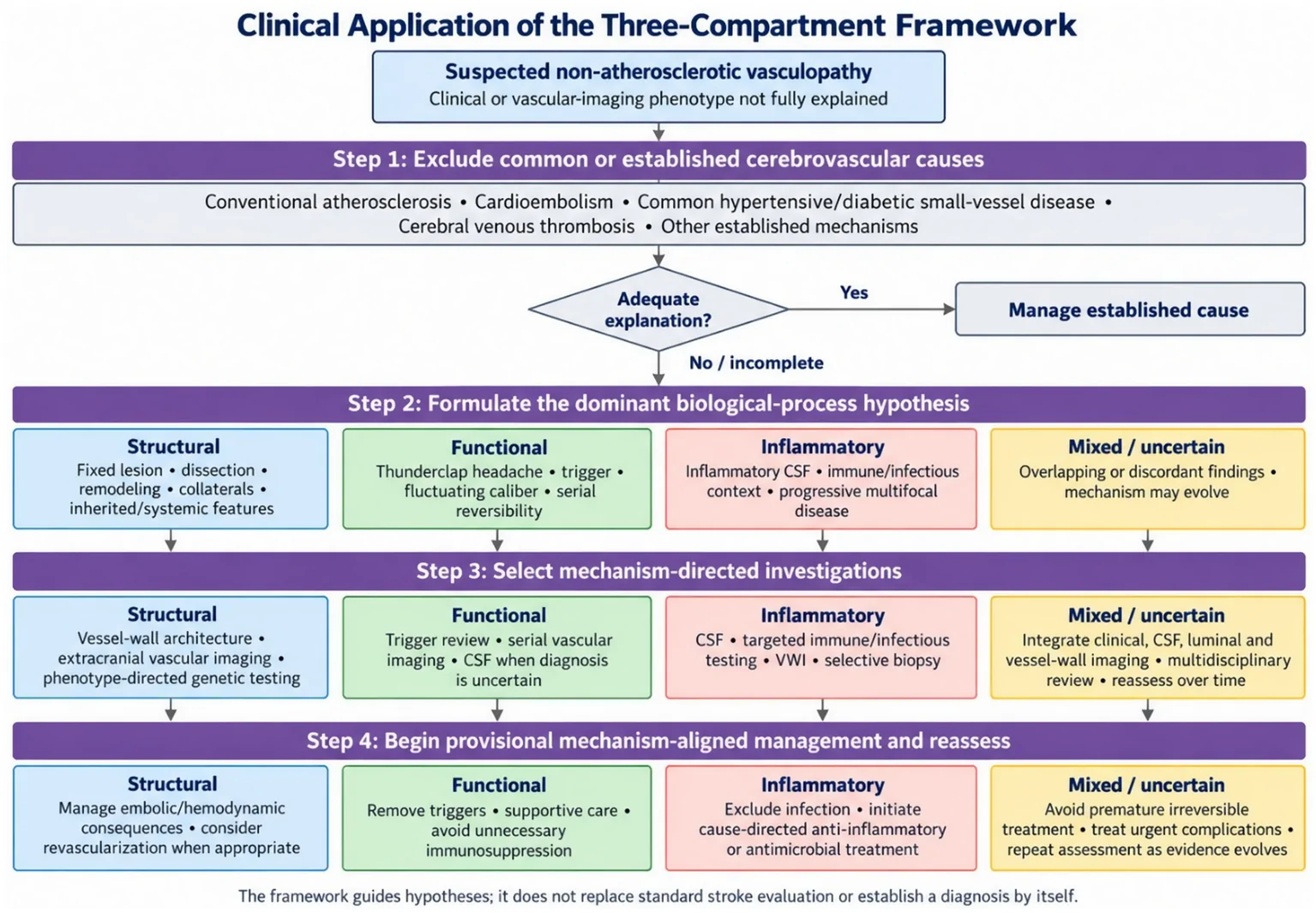
Clinical application of the three-compartment framework. Standard evaluation for common and established cerebrovascular causes should precede application of the framework. When these causes do not adequately explain the presentation, structural, functional, inflammatory, and mixed or uncertain biological-process hypotheses guide subsequent investigations and provisional management. The mixed/uncertain pathway recognises that mechanisms may coexist or evolve and emphasises integrated assessment and diagnostic reassessment over time. The framework supports clinical reasoning but does not replace standard stroke evaluation or independently establish a diagnosis.

Multifocal intracranial stenoses represent a luminal imaging phenotype rather than a specific diagnosis. Conventional angiographic classification identifies the distribution and severity of narrowing but may not reliably distinguish reversible vasoconstriction from inflammatory vascular injury, infection-associated arteriopathy, dissection, or progressive structural remodelling. The three-compartment framework converts this shared phenotype into competing biological hypotheses that guide the sequence of further investigations and initial management.

A predominantly structural hypothesis would be favoured by a fixed or progressively remodelling lesion, intramural hematoma or intimal abnormality, characteristic collateral formation, extracranial vascular involvement, or a relevant familial or syndromic phenotype. Further evaluation may include fat-suppressed arterial wall imaging for dissection, assessment of arterial remodelling and collateral networks, screening of other vascular beds, and phenotype-directed genetic testing for inherited arteriopathies or moyamoya-associated disorders.

A predominantly functional hypothesis would be favoured by recurrent thunderclap headache, exposure to vasoactive triggers, normal or near-normal CSF, and reversibility of arterial narrowing on serial imaging, supporting RCVS. Evaluation would emphasise trigger identification, interval vascular imaging, and assessment for complications such as convexity subarachnoid haemorrhage, ischaemic stroke, or PRES. Initial management would include withdrawal of potential precipitating agents, symptomatic treatment, and careful blood-pressure management; glucocorticoids should be avoided because they may worsen RCVS.

A predominantly inflammatory hypothesis would be considered in the presence of a progressive or relapsing neurological course, inflammatory CSF, systemic inflammatory features, relevant infectious exposures, or supportive vessel wall MRI abnormalities. This pathway prioritises CSF examination, targeted infectious and autoimmune investigations, and vessel wall MRI. Brain or leptomeningeal biopsy may be considered when primary angiitis of the central nervous system remains suspected despite inconclusive non-invasive testing. Vessel wall enhancement should be interpreted as supportive rather than independently diagnostic, and infection should be reasonably excluded before immunosuppressive treatment.

Thus, the framework does not assign a diagnosis or treatment from angiographic appearance alone. Its added value is to determine whether the next discriminating step should be serial imaging for reversibility, CSF and immune or infectious evaluation, detailed structural imaging, or genetic investigation, while reducing the risk of premature treatment, particularly empiric immunosuppression in a functional vasculopathy.

### When to suspect non-atherosclerotic vasculopathy

NAV should be suspected when stroke presents with features atypical for conventional atherosclerotic disease, such as in younger patients, in recurrent or unexplained stroke, in the presence of atypical vascular imaging patterns, or when stroke is accompanied by systemic, genetic, or connective tissue features. Figure 4 summarises these red flags using the acronym “VASCULOPATHY.”

**Figure 4:**
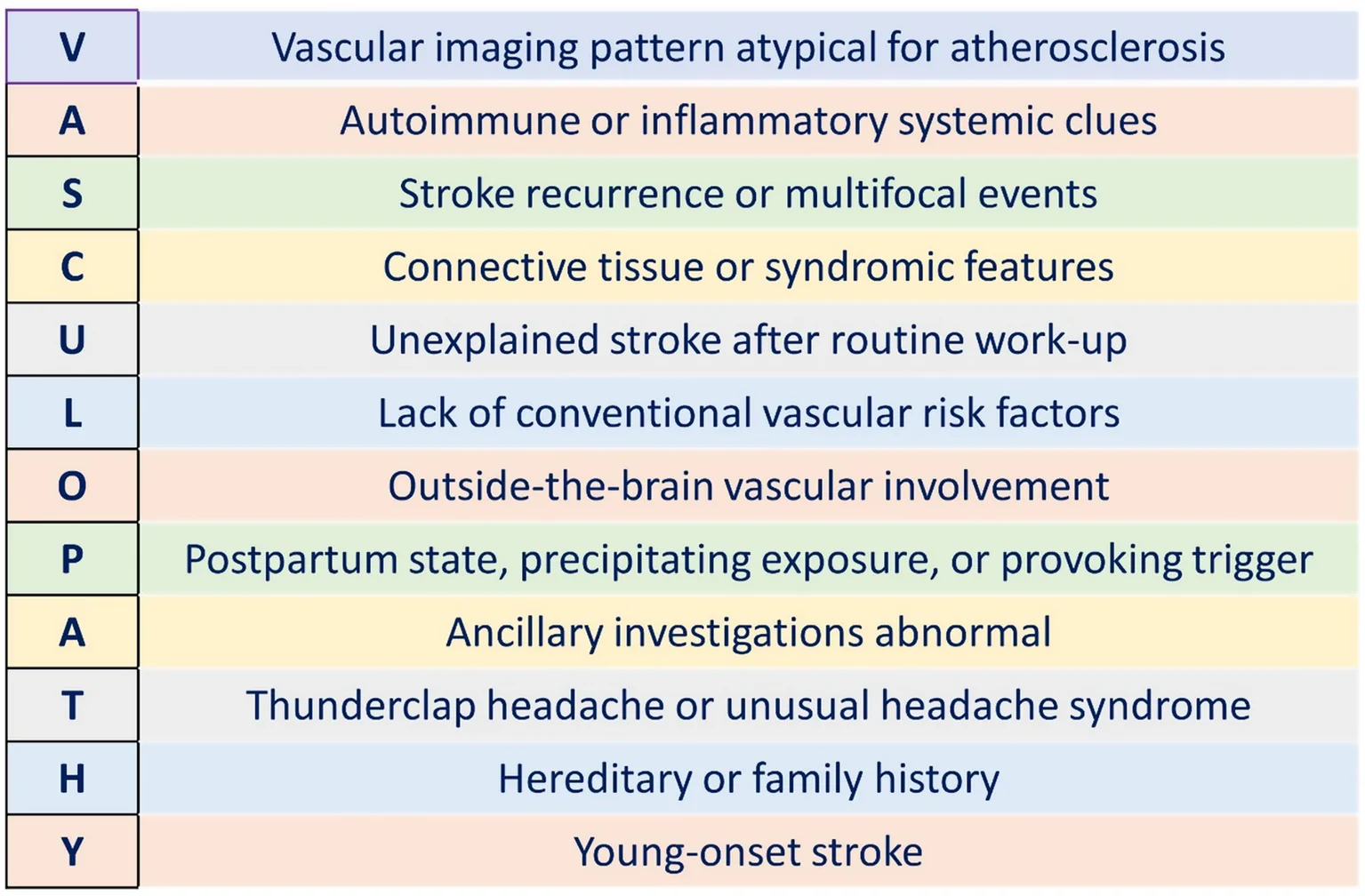
“Vasculopathy” mnemonic for recognising possible non-atherosclerotic vasculopathy. This author-derived mnemonic is intended solely as a clinical memory aid to prompt consideration of non-atherosclerotic vascular disease. It has not been formally derived or prospectively validated and should not be interpreted as a diagnostic criterion, screening instrument, prediction score, or substitute for clinical assessment.

### Evidence gaps and future considerations

Important gaps remain in NAV because most evidence is observational and diagnostic frameworks are not standardised. Future priorities include prospective multicentre registries with harmonised phenotyping, validation of biomarker panels, pragmatic trials in RCVS and PCNSV using standardised outcomes, and standardisation of VWI acquisition and reporting. Implementation-focussed studies should also evaluate scalable diagnostic pathways, including simplified imaging algorithms and point-of-care biomarkers, particularly in resource-limited settings.

## Conclusion

This framework provides a practical biological lens for understanding how NAV arise, evolve, and overlap. By linking the dominant biological process underlying the vascular phenotype to imaging, biomarkers, and treatment, it may improve diagnostic reasoning and support a more mechanism-oriented approach to stroke evaluation.
