# Supplementary material for: Non-atherosclerotic vasculopathies in stroke: a pathobiology-based framework
Source: Front Neurol. 2026 Oct 26;17:1908716. doi: 10.3389/fneur.2026.1908716 (PMC13746346; doi:10.3389/fneur.2026.1908716)
Supplement: Supplementary file 1 [file Supplementary_file_1.DOCX]

**Supplementary Table 1: Causes of Non-Atherosclerotic Vasculopathies Organized by Pathobiological Mechanism**

| **Structural** | **Functional** | **Inflammatory** |
| --- | --- | --- |
| Cervical artery dissection  Fibromuscular dysplasia  Moyamoya disease  Moyamoya syndrome*  CADASIL (*NOTCH3*)  CARASIL (*HTRA1*)  *COL4A1 / COL4A2* arteriopathy  *ACTA2* angiopathy  *DADA2* associated vasculopathy*  Fabry disease  MELAS  Cerebral amyloid angiopathy  Radiation-induced vasculopathy*  Connective tissue disorders (Ehlers–Danlos, Marfan syndrome)  Carotid web  RCVL-S | RCVS  Postpartum angiopathy  Drug-induced cerebral vasospasm (cocaine, amphetamines, sympathomimetics) | PCNSV  Systemic lupus erythematosus  ANCA-associated vasculitis  Polyarteritis nodosa  Behçet disease  Giant cell arteritis  Takayasu arteritis  Varicella-zoster virus vasculopathy  Tuberculous vasculitis  Syphilitic vasculitis  HIV-associated vasculopathy  Post-infectious arteriopathy |

*Overlap syndromes

**CADASIL = Cerebral Autosomal Dominant Arteriopathy with Subcortical Infarcts and Leukoencephalopathy, CARASIL = Cerebral Autosomal Recessive Arteriopathy with Subcortical Infarcts and Leukoencephalopathy, DADA2 = Deficiency of Adenosine Deaminase 2, MELAS = Mitochondrial Encephalomyopathy, Lactic Acidosis, and Stroke-like episodes, RVCL-S = Retinal Vasculopathy with Cerebral Leukoencephalopathy and Systemic manifestations, RCVS = Reversible Cerebral Vasoconstriction Syndrome, PCNSV = Primary Central Nervous System Vasculitis, ANCA = Anti-Neutrophil Cytoplasmic Antibody, HIV = Human Immunodeficiency Virus.

**Supplementary Figure 1:** **Mechanistic pathway underlying structural vasculopathies**

**Supplementary Figure 2:** **Mechanistic pathway underlying functional vasculopathies**

**Triggers**

- Vasoactive medications (triptans, SSRIs, sympathomimetics)
- Recreational drugs (cocaine, cannabis, amphetamines)
- Postpartum hormonal and autonomic changes
- Emotional or physical stress
- Sudden fluctuations in blood pressure

**Disturbance of Vascular Regulation**

- Endothelial dysfunction
- Sympathetic nervous system overactivity
- Reduced nitric oxide mediated vasodilation
- Increased endothelin-mediated vasoconstriction
- Altered serotonin-mediated vascular signalling

**Vascular Smooth Muscle Response**

- Smooth muscle hyperreactivity
- Impaired cerebrovascular autoregulation
- Transient vasoconstriction and vasospasm

**Dynamic Luminal Change**

- Multifocal segmental arterial narrowing
- Dynamic fluctuations in cerebral blood flow
